# Cardiac 4D phase-contrast CMR at 9.4 T using self-gated ultra-short echo time (UTE) imaging

**DOI:** 10.1186/s12968-017-0351-9

**Published:** 2017-03-31

**Authors:** M. Krämer, A. G. Motaal, K-H. Herrmann, B. Löffler, J. R. Reichenbach, G. J. Strijkers, V. Hoerr

**Affiliations:** 1 Medical Physics Group, Institute of Diagnostic and Interventional Radiology, Jena University Hospital - Friedrich Schiller University Jena, Philosophenweg 3, D-07743 Jena, Germany; 2 Biomedical NMR, Department of Biomedical Engineering, Eindhoven University of Technology, Eindhoven, Netherlands; 3 Institute of Medical Microbiology, Jena University Hospital, Friedrich Schiller University Jena, Jena, Germany; 4 Michael Stifel Center for Data-driven and Simulation Science Jena, Friedrich Schiller University Jena, Jena, Germany; 5 Abbe School of Photonics, Friedrich Schiller University Jena, Jena, Germany; 6 Center of Medical Optics and Photonics, Friedrich Schiller University Jena, Jena, Germany; 7 Biomedical Engineering and Physics, Academic Medical Center, Amsterdam, Netherlands

**Keywords:** 4D PC CMR, Self-Gating, UTE, High Field, Blood flow velocity, Mice

## Abstract

**Background:**

Time resolved 4D phase contrast (PC) cardiovascular magnetic resonance (CMR) in mice is challenging due to long scan times, small animal ECG-gating and the rapid blood flow and cardiac motion of small rodents. To overcome several of these technical challenges we implemented a retrospectively self-gated 4D PC radial ultra-short echo-time (UTE) acquisition scheme and assessed its performance in healthy mice by comparing the results with those obtained with an ECG-triggered 4D PC fast low angle shot (FLASH) sequence.

**Methods:**

Cardiac 4D PC CMR images were acquired at 9.4 T in healthy mice using the proposed self-gated radial center-out UTE acquisition scheme (TE/TR of 0.5 ms/3.1 ms) and a standard Cartesian 4D PC imaging sequence (TE/TR of 2.1 ms/5.0 ms) with a four-point Hadamard flow encoding scheme. To validate the proposed UTE flow imaging technique, experiments on a flow phantom with variable pump rates were performed.

**Results:**

The anatomical images and flow velocity maps of the proposed 4D PC UTE technique showed reduced artifacts and an improved SNR (left ventricular cavity (LV): 8.9 ± 2.5, myocardium (MC): 15.7 ± 1.9) compared to those obtained using a typical Cartesian FLASH sequence (LV: 5.6 ± 1.2, MC: 10.1 ± 1.4) that was used as a reference. With both sequences comparable flow velocities were obtained in the flow phantom as well as in the ascending aorta (UTE: 132.8 ± 18.3 cm/s, FLASH: 134.7 ± 13.4 cm/s) and pulmonary artery (UTE: 78.5 ± 15.4 cm/s, FLASH: 86.6 ± 6.2 cm/s) of the animals. Self-gated navigator signals derived from information of the oversampled *k*-space center were successfully extracted for all animals with a higher gating efficiency of time spent on acquiring gated data versus total measurement time (UTE: 61.8 ± 11.5%, FLASH: 48.5 ± 4.9%).

**Conclusions:**

The proposed self-gated 4D PC UTE sequence enables robust and accurate flow velocity mapping of the mouse heart in vivo at high magnetic fields. At the same time SNR, gating efficiency, flow artifacts and image quality all improved compared to the images obtained using the well-established, ECG-triggered, 4D PC FLASH sequence.

**Electronic supplementary material:**

The online version of this article (doi:10.1186/s12968-017-0351-9) contains supplementary material, which is available to authorized users.

## Background

Quantitative blood flow analysis using time-resolved phase-contrast cardiovascular magnetic resonance (PC CMR) is a valuable tool for studying the cardiovascular system in humans and animals [1, 2]. The non-invasive character of PC CMR enables routine clinical examinations of the hemodynamics of blood flow in patients or monitoring cardiac diseases in sophisticated animal models longitudinally over time [3, 4]. Over the last 15 years, the well-established slice-selective PC CMR techniques [5, 6] have been surpassed by extending imaging protocols to three-dimensions or even time-resolved 4D PC CMR [7–10]. The latter provides a far more comprehensive picture of the blood flow as it contains information about the three-dimensional velocity vector field which is specifically valuable to diagnose or assess, e.g., aortic aneurysms [11] or aortic valve prosthesis function [8]. Still, these methods are so far limited in their application to clinical studies and have not been integrated in clinical routine yet. Furthermore, applying 4D PC CMR to small animal cardiac imaging at high field strengths remains technically challenging and has only recently been implemented by using a Cartesian gradient echo sequence [12]. The technical issues are mainly related to the necessary demand on fast-switching gradients when three-dimensional volume excitation and encoding is desired, the very fast cardiac motion of small rodents and the need of robust and reliable respiratory and cardiac triggering over extended periods of time [12].

One major limitation of the currently established PC CMR techniques are the relatively long echo times (TE) of 2 ms and longer [13], which are a direct consequence of the magnetic field gradient switching required for flow encoding and three-dimensional volume encoding in Cartesian gradient echo sequences. In combination with the fast blood flow in the murine heart this often leads to signal dropouts on the images or erroneous flow velocities due to intravoxel dephasing and turbulent fluctuations, hampering the examination of disturbed blood flow patterns in diseases such as atherosclerosis or vascular stenosis [13–16]. One promising alternative is to apply a 3D ultra-short echo-time (UTE) sequence with radial center-out acquisition and no slice selection or phase encoding gradients [17–19]. Starting with data acquisition from the center of *k*-space allows data sampling with echo times below 1 ms, which results in highly reduced flow artifacts [20]. Several studies have already demonstrated that shortening TE most crucially impacts the accuracy of quantitative blood flow measurements [21–23]. Consequently, both a hybrid 4D UTE flow CMR technique based on a *stack-of-stars* approach as well as a spiral flow imaging method have been proposed recently [24, 25]. These techniques, however, have so far only been evaluated at low field strengths. In addition, they still require 3D slab selective excitation and rewinding or use standard 2D multi-slice acquisitions, which are reformatted to 3D datasets potentially leading to signal discontinuities in slice direction. The temporally extended selective excitation still results in echo times well above 1 ms and extended repetition times of more than 5 ms.

A second crucial drawback of currently implemented 4D PC CMR techniques is the long scan time due to the time-resolved sampling of a complete three-dimensional volume and the lower SNR when measuring small animals. Together with the required external cardiac gating of the MR system by using electrocardiogram (ECG) electrodes and the additional respiratory gating, measurement times can easily take up to an hour or longer for a single 4D PC CMR data set of the whole murine heart [12, 26]. A major reason responsible for these long acquisition times is the limited applicability of standard acceleration methods such as GRAPPA [27] or SENSE [28] at high field small animal scanners. Due to the small object geometries receive coils for mice typically feature only very few receive channels allowing only for moderate acceleration to be applied. With very small voxel sizes in the range of 200 μm × 200 μm × 200 μm the typical SNR loss accompanied by the application of acquisition acceleration is also often not acceptable. In addition, ECG gating requires prolonged preparation time of the animals and often suffers from interferences with the magnetic and radio-frequency fields [29, 30], which causes unreliable or unstable trigger signals. On the other hand, applying self-gating strategies by using e.g. additional navigator data periodically acquired in an additional navigator slice [31–34], would only further increase the already very long scan time of 4D PC CMR. To overcome this problem efficient self-gating approaches, i.e. extracting the gating signal from each measurement readout with no recording of additional navigator data [32, 35–37], would be advantageous for a time-resolved 4D UTE acquisition.

In this work we present for the first time a 4D PC UTE acquisition strategy that uses the shortest possible echo- and repetition-times in combination with an efficient self-gating navigator signal extracted from the oversampled center of *k*-space. The proposed technique was validated in both flow phantoms and healthy mouse hearts. Image quality and quantitative flow analysis results were furthermore compared with those obtained with a standard ECG-gated Cartesian 4D PC FLASH sequence on a small-animal MR system.

## Methods

### CMR measurements

All measurements were performed with a 9.4 T Bruker BioSpec USR 94/20 MR scanner with a horizontal bore of 20 cm, a gradient system of 660 mT/m and ParaVision 6.0.1 operation software (Bruker BioSpin, Ettlingen, Germany). For the acquisition of data in vitro, a vendor supplied 40-mm-diameter mouse body quadrature volume coil was used. In vivo experiments were conducted in healthy mice (male, C57BL/6 J, 6 months old) with a vendor supplied two-channel quadrature cryoprobe. Image reconstruction, processing and analysis were performed offline using MATLAB (The Math Works, Natick, MA, USA).

### CMR pulse sequences

**Fig. 1:**
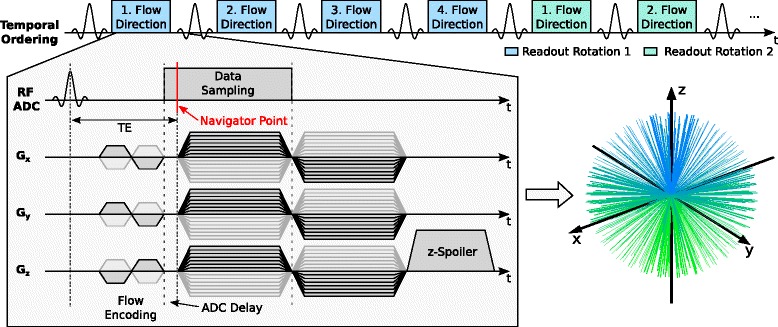
Sequence diagram of the proposed flow encoded time-resolved 4D UTE acquisition indicating the temporal ordering of the flow encoding directions, readout and flow encoding gradient timing. The navigator location obtained from the center of *k*-space is indicated, as are the ADC delay, constant spoiler gradient in z-direction as well as the resulting 3D UTE center-out *k*-space encoding. To realize a cone-by-cone center-out UTE sampling; the readout gradient and dephaser strengths were modulated in the x, y and z directions according to [19], resulting in a 3D k-space encoding which starts in z-direction (*blue color*) and sampling cone by cone towards the negative z-direction (*green color*)

A vendor supplied static center-out cone-by-cone three-dimensional UTE imaging sequence was modified by including a four-point Hadamard encoding scheme [38, 39] for flow velocity encoding prior to the readout gradient in all three spatial dimensions. Geometrically, the cone-by-cone trajectory divides the sampled spherical surface into circles of constant latitude on which all readouts are distributed with uniform distances between readout endpoints so that Nyquist sampling is fulfilled [19]. The four flow-encoding directions were acquired successively for the same readout orientation before acquiring the four flow encoding directions of the next radial readout orientation (Fig. 1). Data sampling during ramp-up of the readout gradient was employed to achieve the fastest possible sampling without having to switch any pre-dephasing gradients. To extract a stable navigator signal from the center of *k*-space of the non-selectively excited three-dimensional imaging volume two crucial modifications were made to the imaging sequence. First, a constant z-spoiling was added because one major source for unwanted contamination of the sensitive navigator signal is the remaining magnetization from previous excitations. Since the three-dimensional center-out radial acquisition changes the readout rotation angles with a short repetition time (TR), unpredictable interferences with the k-zero navigator signal can occur without proper spoiling. The implemented z-spoiling consisted of readout rewinder to the center of *k*-space and application of a constant spoiling gradient in z-direction following each read-out. This simple spoiling scheme causes identical spoiling moments for all readouts independent of their rotation angle. The second source for artifacts in the navigator signal originates from digital sampling artifacts affecting the first data points in the ADC [19]. Unlike the majority of MR imaging sequences, the UTE acquisition starts with the highest signal at the beginning of the ADC activity, which may lead to sampling and digital filtering artifacts in the first sampling points. To allow for sufficient time for the ADC signal to stabilize, the ADC was switched on 100 μs prior to the start of the readout gradient and data points sampled during this time interval were discarded during image reconstruction. The gradient and data sampling scheme of our self-gated time-resolved 4D PC UTE imaging approach is presented in Fig. 1. The shortest possible achievable echo time with this sequence was 0.52 ms, which is primarily limited by the duration of the flow encoding gradients of 0.4 ms.

To assess the quality of the flow measurements performed with this sequence, data were also acquired with a vendor supplied, prospectively triggered Cartesian 4D PC FLASH sequence. The Cartesian 4D PC FLASH sequences used 3D slab selective excitation followed by four-point Hadamard encoding, 3D linearly ordered spatial encoding, single k-space line acquisition and constant spoiling.

### Self-gating and image reconstruction

An intrinsic navigator signal was obtained from each acquired read-out by taking the last data point sampled just before the start of the readout gradient. Depending on the measurement conditions and quality of the navigator signal either magnitude or phase information was manually selected from one of the receiver coil channels. The manual user interaction involved looking at the phase and magnitude of the navigator data separately for all receiver coil channels and subjectively selecting the navigator function which showed the most distinct peaks. With the used cryoprobe having only two receiver channels this manual interaction did typically take not more than 5 min. After selection of the best navigator data the resulting navigator function was filtered using a Savitzky-Golay smoothing filter [40] with a width of 90 ms and a second order degree. The Savitzky-Golay filter essentially performs local fitting of the navigator data to a second degree polynomial in sub-sets of adjacent data points. Peak detection by employing first-order difference information was used to detect cardiac cycle onsets in the navigator function. Regarding respiratory motion, the input navigator function was smoothed with a broader Savitzky-Golay smoothing filter with a width of 400 ms followed again by peak detection. Around each respiratory onset an asymmetric exclusion window of 250 ms duration was placed, within which all measured data were discarded and not used for image reconstruction. The parameters of the smoothing filter and the exclusion area was based on the average respiratory and cardiac cycle patterns for mice used in the experiment. To reconstruct consecutive cardiac cine frames a sliding-window [41] was applied with window width of 15.5 ms and a frame-to-frame shift of 3.1 ms. A total of 48 cine frames spanning 150 ms were reconstructed using this technique. After binning the radial readouts to the corresponding cardiac cycles and flow encoding directions, separate 3D volumes were reconstructed and subsequently combined to a complete 6D (x, y, z, time, flow velocity, coil channel) volume. The radial trajectory was measured using a vendor supplied calibration scan [42], which acquires the trajectory through *k*-space in all three spatial dimensions. To ensure that the measured trajectory is identical to the actually used imaging gradients both the additional ADC delay as well as the ramp-up shape of the readout gradient were included in the trajectory calibration.

Image reconstruction of the 3D volume was performed using state-of-the art regridding with iterative sampling density estimation and an optimized kernel [43]. Regridding was performed onto a trifold oversampled grid with additional zero filling by a factor of two. After gridding a three-dimensional Fast-Fourier-Transform (FFT) was performed followed by a phase offset corrected receive channel combination. For automated detection of the receive channel phase offsets the magnitude of the complex sum of all receive channels was iteratively maximized using an unconstrained nonlinear optimization with the phase offsets as variables. Following matching of the phase offsets, the receive channel combination of the magnitude and the phase was computed by the square root of the sum-of-squares signal and the phase of the sum of the complex values, respectively. Receiver channel combination was performed prior to the computation of the velocity fields in order to avoid increased flow velocity noise in the transition regions between the receive channels. Image reconstruction of the vendor supplied Cartesian reference sequence was also performed offline using MATLAB with the same reconstruction steps as for the UTE sequence (except for self-gating and regridding), including *k*-space zero filling by a factor of two and phase offset corrected receive channel combination. In all in vivo measurements the maximum encoded flow was 120 cm/s which was set very close to the expected maximum flow in mice to maximize sensitivity. In some cases, where the highest flow was directed exactly along a single axis, phase unwrapping was performed on the calculated flow velocity maps [44]. All image reconstruction and processing was performed offline on a high performance computation system with 64 CPU cores and 512 GB of memory. Overall reconstruction time of the UTE magnitude images and flow velocity maps was approximately 6 h per data set, with the majority of time spend on 3D gridding (3 h) and receive channel combination (1 h) as well as raw data loading and storage of the reconstructed data (1 h). Except for manual navigator selection all remaining image reconstruction was fully automated without any user interaction.

### In vitro flow measurements

In order to validate the flow velocities obtained from the time-resolved 4D PC UTE imaging sequence phantom measurements were performed using a home-built flow phantom containing four tubes with a diameter of 3 mm placed inside a water-filled cylindrical acrylic glass body with a diameter of 30 mm [12]. The tubes were interconnected in an alternating fashion and attached to a peristaltic pump (Medorex e.K., Hörten-Hardenberg, Germany), and flow measurements were performed using different pump rates. To compensate the pulsation of the flow caused by the peristaltic pump a series of air filled acrylic glass cylinders was integrated between the pump and the flow phantom, acting as a compressible medium and effectively homogenizing the flow. As gold-standard a vendor supplied Cartesian three-dimensional FLASH based flow mapping imaging sequence (Flowmap, Bruker BioSpin, Ettlingen, Germany) was used and the results were compared to those of the 4D PC UTE sequence. Applying a four-point Hadamard encoding scheme in both sequences, all three spatial velocity components were encoded. The Cartesian FLASH sequence used a 25% readout Partial Fourier factor to minimize the echo time and was performed with the following acquisition parameters: 2.5 ms TE, 6.5 ms TR, 1 h 25 min total acquisition time (TA), 4 averages and 3D slab-selective sinc-pulse excitation with a 10° flip angle and a pulse duration of 1.4 ms. For the 4D PC UTE sequence the following parameters were used: 0.5 ms TE, 5.0 ms TR, 1 h 45 min TA, 2 averages and a non-selective hard-pulse excitation with a 10° flip angle and a pulse duration of 40 μs. Both imaging sequences acquired data with a FOV of 32 × 32 × 32 mm^3^ and an acquisition matrix size of 200 × 200 × 200 using a 40-mm-diameter quadrature volume coil with the cylindrical flow phantom placed in the coil center. To account for the varied pump rates of 200, 400, 600 and 750 ml/min, the strength of the flow encoding gradients was adjusted to a maximum flow (v_enc_) of 50, 90, 150, 200 cm/s in both sequences, respectively. Flow volumes (ml/s) were determined in all four tubes of the flow phantom from Regions-of-interest (ROIs) drawn around the tubes. Additionally, profile lines were drawn through the center of each tube to compare the flow profiles in the tubes.

### In vivo flow measurements

Blood flow measurements were performed in a group of ten healthy mice using either the Cartesian FLASH sequence with ECG- and respiratory gating as gold-standard (*n* = 5) or the proposed time-resolved 4D PC UTE imaging sequence with self-gated acquisition (*n* = 5). A mixture of 1.5 to 2.5% isoflurane and O_2_ was used for inhalation anesthesia during the measurements. For optimum positioning of the mouse heart with respect to the tight cryoprobe the animals were positioned on their back with the thorax and heart facing up towards the coil. The total time in the MR scanner, including positioning, ECG setup, localization and shimming, was kept below 3 h. During all experiments body temperature and respiration were monitored and the latter kept at steady rates as best as possible. Body temperature was maintained by using the measured body temperature in an automated feedback loop for adjusting the temperature of a heating pad below the animals. All animals recovered well after the examination.

All measurements were performed with a FOV of 22 × 22 × 22 mm^3^ to cover the whole heart and an acquisition matrix size of 96 × 96 × 96, resulting in an isotropic resolution of 230 μm. To minimize the TE of the Cartesian FLASH sequence, a 25% readout Partial Fourier acquisition was used, resulting in a TE of 2.1 ms and a TR of 5.0 ms with an acquisition bandwidth of 85 kHz. For the UTE acquisition both TE and TR could be shortened to 0.5 ms and 3.1 ms, respectively, while using an acquisition bandwidth of 150 kHz. Both sequences used an excitation flip angle of 10° realized with a 40 μs hard-pulse excitation in the UTE sequence and a 3D slab-selective sinc-pulse excitation in the FLASH sequence. With the Cartesian FLASH sequence 20 frames of the cardiac cycle were acquired with a planned TA of 50 min. However, due to the ECG and respiratory gating the effective TA lasted, depending on the gating efficiency, between 1 h35 min and 1 h55 min. For the self-gated UTE sequence a fixed TA of 1 h 58 min was used during which 20 repetitions, each containing 28733 radial readouts per flow encoding direction, were acquired. For all measurements in vivo the flow encoding gradients were adjusted to a v_enc_-value of 120 cm/s.

Signal-to-noise ratios (SNR) and contrast-to-noise ratios (CNR) were calculated from the reconstructed magnitude data. ROIs were defined on a cardiac short axis slice in the left ventricular cavity and the myocardium for all reconstructed repetitions and the corresponding SNR and CNR were calculated as average values. For a qualitative description of the computed flow velocity maps, the flow velocity noise was calculated as the standard deviation of the absolute value of the flow velocity in the ROIs of the myocardium. For further comparison, the maximum blood flow velocity values (v_max_) were calculated in the pulmonary artery (PMA) and the ascending aorta (AAO). The gating efficiency was calculated from the planned and the actual measurement time for the Cartesian FLASH sequence and from the number of readouts included in the image reconstruction for the UTE sequence divided by the number of measured readouts. The error rates of wrongly detected cardiac onsets during self-gating were obtained by calculating the heart rates within each respiratory cycle, counting the deviations from the average heart rate and comparing the result to the total number of cardiac cycles.

Animated cine images of the flow in the left and right ventricle showing the anatomic magnitude, flow and an overlay of flow and anatomic magnitude are available as Additional files 1, 2, 3 and 4. 



**Additional file 1: FLASH-LV.** (AVI 2487 kb)




**Additional file 2: FLASH-RV.** (AVI 1898 kb)




**Additional file 3: UTE-LV.** (AVI 4744 kb)




**Additional file 4: UTE-RV.** (AVI 3549 kb)


## Results

### In vitro flow measurements

**Fig. 2:**
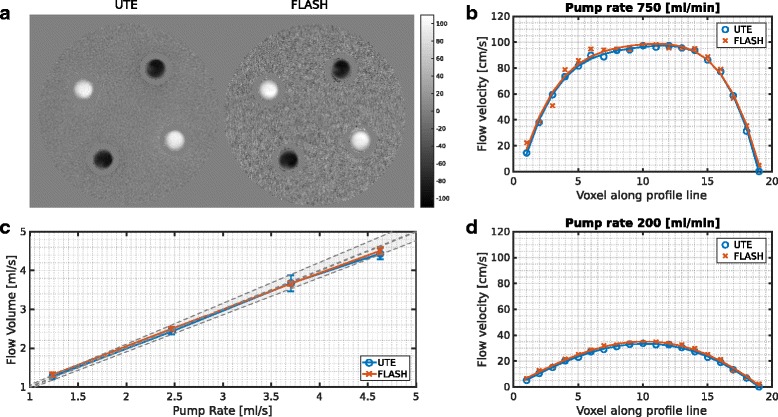
Axial view of the central slice through the flow phantom acquired with the 4D PC UTE and the Cartesian PC FLASH sequence (**a**), corresponding flow profiles of one tube for a pump rate of 750 ml/min (**b**) and 200 ml/min (**d**) as well as the flow volume averaged over all tubes for different pump rates (**c**). In (**c**) the *gray line* represents the pump rate the pump was set to and the corresponding error region for the approximate accuracy that can be expected from the used pump. The axial flow velocity maps shown in (**a**) were masked based on the magnitude image to exclude noise contributions outside of the phantom and in the border areas of the glass phantom

To validate the 4D PC UTE sequence we performed flow measurements on a flow phantom adjusted to different pump rates and compared the results to those obtained from the vendor supplied Cartesian FLASH sequence (Fig. 2). With both sequences the four tubes of the phantom were clearly identified on an axial slice cutting the tubes perpendicularly with two tubes showing positive and two tubes showing negative flow on the maps (Fig. 2, a). Higher noise was clearly noticeable in the static water filled background area of the phantom for the Cartesian FLASH sequence. The velocity profiles across the tubes for two different pump rates, corresponding to the range of laminar and turbulent flow (Fig. 2, b and d), showed close agreement between both sequences, with the Cartesian FLASH sequence yielding slightly higher flow velocities. This became also evident when determining the flow volume for different pump rates and averaging over all four tubes (Fig. 2, c). For all pump rates, the Cartesian FLASH sequence provided slightly higher flow velocities with an average deviation from the UTE sequence of 2.2 ± 1.6%. For both sequences the flow volumes did coincide with the pump setting.

### In vivo flow self-gating

**Fig. 3:**
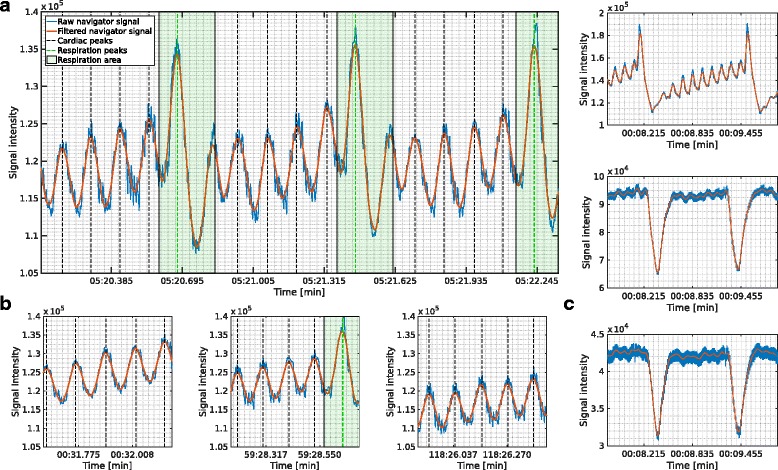
Various forms of the navigator signal extracted from the magnitude of the *k*-space center before (*blue*) and after (*red*) filtering. The *black dashed lines* and the *green dashed line* indicate the detected cardiac and respiratory onsets, respectively. Also shown are the respiration windows within which all data are discarded and excluded from image reconstruction. Displayed is the navigator signal in (**a**) over three respiratory cycles, in (**b**) from different time points during the 2 h measurement (*left to right*) and in (**c**) with ADC delay and z-spoiling (*top*), with ADC delay and no z-spoiling (*middle*) and without ADC delay and no z-spoiling (bottom)

For 4D PC UTE flow measurements in vivo a reliable navigator signal was extracted from the *k*-space center sampled by each readout. Exemplary navigator signals obtained from the *k*-space center magnitude are shown in Fig. 3. Due to the constant z-spoiling and by discarding of digital filtering artifacts in the first ADC sampling points a clear navigator signal showing both cardiac and respiratory motion could be extracted (Fig. 3, a). Although respiration is also visible without z-spoiling and ADC delay (Fig. 3, c, bottom) cardiac motion is obfuscated by high frequency fluctuations in the navigator signal. This high frequency noise in the navigator function is successfully reduced by the introduction of the ADC delay (Fig. 3, c, middle) and removed to large parts by also adding z-spoiling (Fig. 3, c, top). Following further processing with a smoothing filter the signal was well suited for the automatic detection of both cardiac and respiratory signal modulations required to reconstruct images corresponding to different cardiac frames. Definition of exclusion windows around all respiratory peaks was necessary to avoid not only the detection of cardiac onsets occurring during breathing but also to prevent detected cardiac peaks shifting into zones of respiration during the sliding-window reconstruction. The average gating efficiency of the self-gated 4D UTE sequence was 61.8 ± 11.5% while the Cartesian FLASH sequence showed a smaller gating efficiency of 48.5 ± 4.9% due to the active ECG and respiratory gating. Because retrospective gating is used with the UTE sequence and 3D *k*-space data are collected by repeated but continuous acquisition, some radial rotation angles are used multiple times for a certain combination of the reconstructed flow encoding direction and time frame while others are not selected. While the actively triggered Cartesian FLASH sequence naturally had a *k*-space coverage of 100%, the coverage was smaller with the UTE sequence as only 85.1 ± 8.4% of radial rotation angles required for a full k-space coverage were measured. Over the total measurement duration of 2 h the navigator signal was stable (Fig. 3, b) and showed an average error rate of wrongly detected cardiac onsets of 4.3 ± 2.4%.

### In vivo flow measurements

**Fig. 4:**
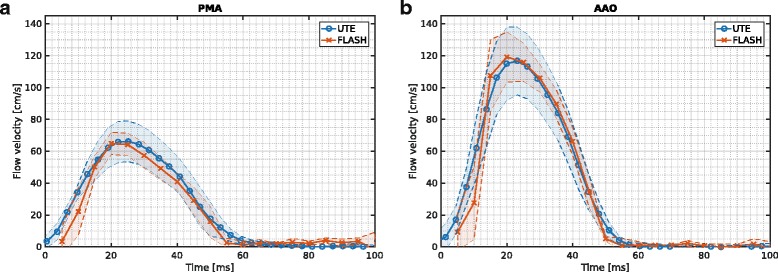
Flow velocity-time course averaged over five animals in the pulmonary artery (PMA) (**a**) and ascending aorta (AAO) (**b**) for the 4D PC UTE (*blue*) and the Cartesian FLASH (*red*) sequence. The colored error bands indicate the standard deviation over all animals

The peak blood flow velocities in the AAO were 132.8 ± 18.3 cm/s and 134.7 ± 13.4 cm/s for the UTE and Cartesian FLASH sequence, respectively. Results obtained for the PMA were also similar for both sequences with respective values of 78.5 ± 15.4 cm/s (UTE) and 86.6 ± 6.2 cm/s (FLASH). The averaged temporal velocity waveforms over all animals in the PMA and AAO, revealed good conformity in amplitude and temporal evolution (Fig. 4) for both sequences. A higher temporal resolution due to the shorter TR and due to a greater number of time frames that could be reconstructed retrospectively was achieved for the UTE sequence compared to the FLASH reference.

**Fig. 5:**
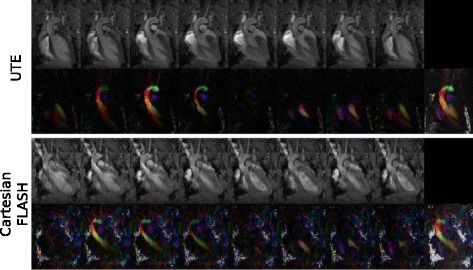
Anatomic magnitude images and color-coded flow velocity images of a coronal single slice through the left ventricle for the 4D PC UTE and the Cartesian FLASH reference sequence. Shown from left to right are 8 frames of the cardiac cycle. The flow velocity image in the ninth column is the maximum intensity projection over all frames. For illustration the flow velocity maps are color encoded and are scaled between the velocities of 0 and 120 cm/s, showing the absolute blood velocity for flow from top to bottom in *red*, from left to right in *green*, and for through-plane flow in *blue*. Note that due to the long measurement duration the images of the two sequences were obtained from different measurement sessions and mice

Substantial differences in image quality were noticed, however, when comparing the magnitude images of the cardiac anatomy for both sequences (Fig. 5). The reconstructed UTE images were free of major artifacts with SNR (left ventricular cavity: 8.9 ± 2.5, myocardium: 15.7 ± 1.9) and CNR (7.8 ± 2.4) values that were nearly a factor of two higher than the values obtained from images acquired with the Cartesian FLASH reference sequence (Table 1). In comparison to the UTE images, the Cartesian FLASH sequence resulted in diffuse artifacts overlaying the whole image with changing shapes and positions in each time frame. The image quality directly affected the calculation of the flow velocity maps and the Cartesian FLASH sequence showed substantially more noise both in the vessels and in static areas and tissue such as the myocardium where no flow was supposed to occur. The standard deviation of the flow velocity in the myocardium showed a substantially reduced flow noise of 3.2 ± 1.1 cm/s with the UTE sequence compared to the Cartesian FLASH reference which resulted in a value of 10.8 ± 6.8 cm/s.

**Table 1:** Quality parameters of images and flow velocity maps

	SNR LV	SNR MC	CNR	Gating efficiency [%]	*k*-space coverage [%]	Flow velocity noise [cm/s]	Peak flow velocity AAO [cm/s]	Peak flow velocity PMA [cm/s]
4D PC UTE (*n* = 5)	8.9 ± 2.5	15.7 ± 1.9	7.8 ± 2.4	61.8 ± 11.5	85.1 ± 8.4	3.2 ± 1.1	132.8 ± 18.3	78.5 ± 15.4
4D PC Cartesian FLASH (*n* = 5)	5.6 ± 1.2	10.1 ± 1.4	4.5 ± 2.2	48.5 ± 4.9	100.0 ± 0.0	10.8 ± 6.8	134.7 ± 13.4	86.6 ± 6.2

Quality parameters of images and flow velocity maps averaged over all performed measurements: signal-to-noise ratio (SNR), left ventricular cavity (LV), myocardium (MC), contrast to noise ratio (CNR), pulmonary artery (PMA) and ascending aorta (AAO)

**Fig. 6:**
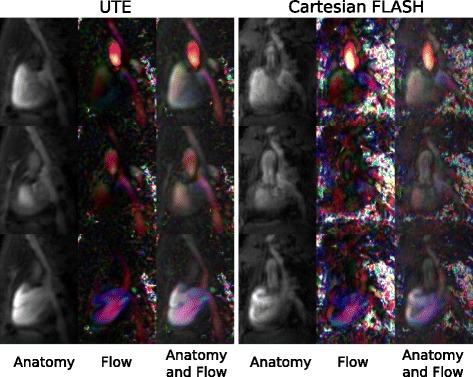
Anatomic magnitude, color encoded flow velocity images and overlay of magnitude and flow images showing the right ventricle for the 4D PC UTE and the Cartesian FLASH sequence. Three frames of the cardiac cycle are displayed from top to bottom, respectively. For illustration the flow velocity maps are scaled between velocities of 0 and 60 cm/s showing the absolute blood velocity for flow from top to bottom in *red*, from left to right in *blue*, and for through-plane flow in *green*

A reformatted slice of the velocity encoded 4D volumes showing the inflow of the right ventricular cavity is presented in Fig. 6. As artifacts in the UTE images were distinctly reduced, the tricuspid valve became clearly visible and was not obfuscated by artifacts as in the Cartesian FLASH reference. The observed increased flow velocity to noise ratio obtained with the UTE sequence (Table 1) is also evident in the shown images. While both superior and inferior vena cava were clearly visible on the magnitude anatomy images and velocity maps, the velocity values in those vessels were only barely distinguishable from the background noise with the Cartesian FLASH sequence. By overlaying the color encoded velocity maps with the magnitude anatomy we were able to demonstrate that with both sequences anatomy and blood flow coincide spatially and temporally.

## Discussion

Our proposed self-gated 4D PC UTE sequence enabled measuring flow velocities of the whole heart in all three spatial dimensions with full temporal coverage of the cardiac cycle in healthy mice. Compared to a vendor supplied Cartesian 4D PC FLASH sequence, image and flow artifacts were substantially reduced accompanied by higher SNR and CNR. In addition, the time consuming and artifact affected active external ECG gating was unnecessary. One of the reasons for the high performance of the UTE technique is the radial center-out 3D acquisition which renders the measurement very robust against motion related *k*-space inconsistencies caused by object motion or small heart rate changes, which are inevitably occurring during long acquisition times. During anesthesia the physiological conditions, such as respiration and heart rate, can usually be kept constant only to a certain extent and ventilation of the animals would be required for better control. Besides the reduction of artifacts in the images acquired by the 4D PC UTE sequence, radial acquisition is also suitable for retrospective motion correction [45] to be applied in future work to correct for small long term subject movements. With a Cartesian line by line sampling in contrast, even small changes of the object will cause inconsistencies in the 4D *k*-spaces that easily lead to artifacts affecting the whole image.

### Acquisition time

Even though the applied center-out 3D UTE sampling technique requires more readouts by a factor of π to fill 3D *k*-space completely compared to the prospectively gated Cartesian FLASH sequence, the overall measurement time was the same for both sequences. As our results demonstrate, the increased amount of required readouts can be compensated easily by shorter echo- and repetition-times in combination with an efficient retrospective gating strategy. Specifically, by using self-gated navigator signals based on the information obtained from the oversampled center of *k*-space, no additional time consuming data acquisition is required. With the reduced TR and the retrospective gating twice as many frames of the cardiac cycle can be acquired in the same acquisition time. Moreover, animal preparation and handling can be accelerated substantially, as the placing of ECG electrodes can be avoided completely. This does not only reduce stress for the animals but also allows for potentially longer experiment durations in the scanner. Although the acquisition time of 2 h per data set is long compared to 4D flow measurements performed in humans it has to be pointed out that having mice under anesthesia for 2 h was unproblematic for all animals measured in our experiments. In addition isotonic saline can be applied supportively to healthy and diseased animals before scanning to prevent dehydration. Finally, with the 4D UTE acquisition providing better SNR in both anatomical images and reconstructed flow velocity maps, acquisition times can be potentially further shortened. When translating the technique to human imaging the better SNR and thus shorter acquisition times should also make the sequence attractive for future translation into the clinics.

One alternative approach for reduction of acquisition time is the vastly undersampled isotropic projection reconstruction (VIPR) approach [46] which has also already been combined with phase contrast based flow quantification (PC-VIPR) [47]. The VIPR technique is often used for angiography and uses very high undersampling factors of 10 to 60. However, with VIPR only projections of the whole 3D volume can be reconstructed limiting the possibilities of data analysis. Furthermore, VIRP often requires contrast agents for optimized performance and has so far only been demonstrated with center-in readouts and thus longer echo times.

### Radial trajectory measurement

As has been already demonstrated in the literature [34, 48, 49], precise trajectory measurements are crucial and of utmost importance to obtain good image quality with radial imaging techniques. Since trajectory errors might also influence the calculated flow velocities, we used a vendor supplied calibration [42], which measures the trajectory through *k*-space in all three spatial dimensions based on the actually applied imaging gradients. The calibration was performed prior to each measurement to compensate also for short term fluctuations of the gradient system [50]. Minor remaining trajectory imperfections might be the reason for the overall slightly lower velocity values acquired with the 4D PC UTE sequence (Fig. 2, Table 1).

### Self-gating

The navigator signal extracted from the center of the three-dimensional *k*-space turned out to be very reliable over the long measurement durations. One drawback of the navigator analysis employed in this work, however, was that manual user interaction was required to reconstruct each dataset. Depending on the specific measurement conditions, such as receiver gain, shim settings or the exact animal placement, periodic navigator signal was often more pronounced in either the magnitude or the phase of the *k*-space center and also showed differences in the navigator quality in the receive channels. Although manual user interaction may hamper application of the self-gating technique in routine clinical settings at present and requires development of an automated quantitative post-processing framework in the future, user interaction is certainly acceptable in pre-clinical research.

The ADC delay of 100 μs used in our experiments was preventive to ensure that the signal of the k-space center used for both self-gating and gridding had minimal contaminations from digital filtering artifacts. For future optimizations of the sequence and for a reduction of the TE it should be possible to obtain satisfactory results even with smaller ADC delay values. The ADC delay ultimately depends on the amount of acquired readout points and thus on the acquisition bandwidth. Higher acquisition bandwidths would also allow for acquisition of more readout points in the same time and thus a reduction of the ADC delay.

The temporal frame-to-frame shift of the sliding-window used for self-gating was 1 TR and thus corresponds to the temporal resolution of the gating function. In future work reconstruction of higher temporal resolutions might be possible by reducing the width of the sliding-window or by a more advanced navigator analysis e.g. the interpolation of the self-gating function to a sub TR grid.

### K-space sampling

For the proof-of-principle demonstration of our proposed self-gated 4D PC UTE sequence, image reconstruction was performed without applying any acquisition acceleration or regularized image reconstruction. With an average *k*-space filling of 85.1 ± 8.4%, *k*-space data was only slightly under-sampled, rendering the application of any advanced reconstruction methods unnecessary. Nevertheless, shortening the acquisition time further or increasing spatial resolution the presented 4D PC UTE technique might benefit from applying Compressed Sensing [51], 3D Radial GRAPPA [52] or even a combination of these techniques [53] in order to compensate for larger under-sampling factors. However, it has to be pointed out that it is currently not clear to what extent the application of acceleration techniques in combination with a center-out radial 3D sampling will influence the reliability of the calculated flow maps [49].

In our sequence the 4D UTE sampling is based on a linear cone-by-cone temporal encoding of the radial rotations angles which results in a sufficiently filled *k*-space of 85.1 ± 8.4% as discussed earlier. However, as shown with a recently proposed self-gated 2D PC radial FLASH sequence [49] using golden-angle based rotation angles [54] or the extension to 3D center-out radial sampling [55, 56], *k*-space homogeneity after self-gating can be further increased which consequently allows for further shortening of the total acquisition time.

### Comparison to other techniques

Aside from the differences in the noise of the flow velocity maps, SNR, CNR and image quality, our results show that averaged flow velocities obtained in vivo in larger arteries closely agree between the UTE and the Cartesian FLASH sequences. This is in line with the results from phantom measurements which also revealed identical flow values for both techniques and different velocity ranges. Remaining fluctuations and differences in the measured blood flow velocities in vivo is mostly attributed to the fact that the measurements were performed separately with different mice and on different days. Due to the long scan times for both UTE and Cartesian FLASH imaging, measurements of the same animal on the same day were not feasible.

Compared to other recently proposed non-Cartesian cardiac 4D PC imaging techniques using stack-of-spirals or stack-of-stars [24–26] trajectories, our proposed three-dimensional center-out radial sampling in combination with non-selective excitation slice, allows to fully avoid spatial selection and gradient dephasing. This enables the shortest possible echo times in the order of 0.5 ms, which is only limited by the duration of the flow encoding gradients. Compared to non-Cartesian 4D PC imaging methods using stack-of-spirals [26] and full 3D encoding our proposed radial center-out sampling enables shorter echo-times and avoids some of the limitations of spiral imaging [57]. While the measurement time is certainly longer than that achieved with 4D spiral PC imaging [26] whole heart coverage is realized with the proposed method compared to the acquisition of only a small 3D slab [26]. Compared to previous techniques [24, 25] our reconstructed data is spatially isotropic without any discontinuities. The selection of the appropriate method therefore depends on the desired application, e.g. whole heart flow velocity analysis versus flow velocity analysis in only a small region such as the aortic arch. With the proposed sampling it should in theory, and with respect to the basic gradient timing, also be possible to achieve even shorter repetition times than the currently used 3.1 ms, which would further increase temporal resolution and sampling efficiency. However, with the used 9.4 T small animal CMR system hardware restrictions on how fast different gradient shapes can be transferred to the scanner hardware limited the ultimate repetition time. With other CMR systems, like low field clinical systems, shorter repetitions times can be achieved [19] which might be of advantage for further applications to human cardiac imaging or when using different small animal scanners.

### Limitations of this study

Although more cine frames could be reconstructed with the UTE PC sequence one has to keep in mind that the used sliding-window-reconstruction will cause a slight broadening of the temporal flow velocity profile due to its effect as a moving average filter. With respect to the measured peak velocities, the sliding-window-reconstruction should, however, have only a small effect since the temporal evolution of the peak flow in mice has a peak width of about 25 to 40 ms which is much larger than the width of the sliding-window of 15.5 ms. Moreover, the width of the sliding-window can potentially also be reduced to 9.1 ms if a very accurate evaluation of the temporal flow velocity profile is desired.

In the present study, experiments were performed on healthy mice and it has not yet been investigated if the proposed self-gating based on the oversampled signal of the 3D k-space center will also provide reliable results in mice with altered hemodynamics. However, it has previously been shown that cardiac k-zero self-gated 2D UTE imaging can be performed in mice with myocardial infarction [36]. Even a diseased heart with highly reduced function and only small anatomical changes between diastole and systole still causes large volumes of blood to flow during the cardiac cycle and consequently induces changes in the self-gating signal. Regarding the problem of unstable heart rates it is not clear how exactly an arrhythmia would manifest in the self-gating signal. As has been shown recently, self-gating can be performed in the presence of arrhythmia by detecting self-gating signal abnormalities and excluding the data from image reconstruction [58]. With an appropriate modification of our proposed self-gating analysis detection of arrhythmia is expected to be feasible.

## Conclusion

In conclusion, we have shown that the proposed self-gated 4D PC UTE sequence enables robust and accurate flow velocity mapping of the mouse heart in vivo at high magnetic fields. Due to the radial nature of the acquisition, artifacts in the anatomic magnitude images as well as in the calculated flow velocity maps are reduced when compared to a well-established, ECG-triggered, 4D PC FLASH sequence. In comparison to the Cartesian reference both anatomic SNR and CNR as well as flow velocity SNR are improved at the same time. The used self-gating, based on information obtained from the oversampled k-space center, was very reliable over several hours of measurement time and resulted in an improved gating efficiency when compared to active ECG gating.

## Abbreviations

AAO: Ascending aorta
ADC: Analog to digital converter
CMR: Cardiovascular magnetic resonance
CNR: Contrast-to-noise ratio
ECG: Electocardiogram
FFT: Fast-Fourier-Transform
FLASH: Fast low angle shot
FOV: Field of view
GRAPPA: Generalized autocalibrating partially parallel acquisitions
LV: Left ventricular cavity
MC: Myocardium
PC: Phase contrast
PMA: Pulmonary artery
ROI: Region of interest
SNR: Signal-to-noise ratio
TA: Acquisition time
TE: Echo time
TR: Repetition time
UTE: Ultra-short echo time
